# Mothers’ Difficulties and Expectations for Intervention of Bullying among Young Children in South Korea

**DOI:** 10.3390/ijerph16060924

**Published:** 2019-03-14

**Authors:** Seung-ha Lee, Hyun-jung Ju

**Affiliations:** Department of Early Childhood Education, Chung-Ang University, Seoul 06974, Korea; jhj834@gmail.com

**Keywords:** Bullying, intervention, young children, South Korea, hakkyo-pokryuk, prevention

## Abstract

This study investigated the difficulties of mothers in coping with the bullying of their children and their expectations concerning bullying intervention for young children in South Korea. Twenty mothers with young children were interviewed. Interviews were transcribed in Korean. Nvivo 12 software was used to analyze the data. Four themes emerged: “mothers’ coping strategies”, “problems of interventions”, “expectations of interventions”, and “developmentally appropriate interventions for young children”. Each theme was divided into categories and further into subcategories. Mothers used diverse strategies to intervene when their children were bullied and showed dissatisfaction with the current intervention system. Their expectations for interventions for young children were explained in terms of familial, school, and local/governmental levels. These results emphasized that intervention policies for bullying among young children should be urgently established, and intervention programs need to consider the developmental characteristics of young children.

## 1. Introduction

Bullying is defined as an aggressive behavior that is repeated on a targeted person who has difficulty defending themself [1]. Bullying research has heavily focused on mid-childhood and adolescence; however, studies have shown that bullying clearly exists among young children [2,3,4]. Characteristics of bullying in early childhood differ from those in middle childhood or adolescence; they are less likely to be repeated on a targeted person, and children are less-likely to perceive an imbalance of power. Additionally, the bullying behavior of young children tends to show low stability, especially in terms of the victim role [4,5,6]. Due to the different characteristics of bullying in early childhood, researchers have suggested diverse terms such as unjustified aggression [5] or precursory bullying [6]. Kirves and Sajanieme [7] reported that 12.6% of daycare children were directly involved in bullying and even 3-year-old children were able to identify bullying and distinguish bullying from quarrelling.

### 1.1. Coping Strategies of Young Children

Young children cope with bullying in varied ways. In Monks et al., 4–6-year-old children coped with bullying by most frequently telling an adult, followed by fighting back, getting a friend to help, walking away, crying, and giving something up to the aggressor [5]. Similarly, Reunamo and colleagues interviewed 761 young children about coping strategies in bullying situations [8]. Their results showed that among the children’s responses, 70% engaged in participative strategies, that is, doing something to remedy the situation (e.g., telling the teacher, defending oneself, asking to play together, or settling the quarrel, etc.). Sixteen percent showed withdrawal strategies (e.g., going away, telling a teacher or mother, leaving, playing alone, or crying), 6.8% of uncertain strategies (e.g., not knowing what to do, or doing nothing), and 5.1% of dominant strategies (e.g., pushing bullies far away, hitting or teasing back) were shown. Only 1.6% of the children’s responses did not care about the bullying (e.g., “I don’t care”, “I will be in peace”). Therefore, the majority of young children asked for support from adults and some children used ineffective strategies (e.g., fighting back or passive reaction). These results suggest that adults need to play a role in stopping bullying among young children. In other words, young children should be guided to employ appropriate and effective coping strategies by adults.

### 1.2. Intervention Program for Bullying in Early Childhood

Intervention programs have been developed to prevent bullying among young children [9,10,11]. These have generally focused on increasing the social emotional competence and assertive skills of children, and training teachers to cope with children’s bullying. One of the most well-known programs is the Bernese Prevention Program against Victimization (Be-Prox) developed by Alsaker in Switzerland [9]. It focuses on enhancing teachers’ competence in managing bullying among young children. This program increases teachers’ sensitivity to bullying, enhances their skills, and helps establish principles for positive peer interactions. Additionally, parental involvement is encouraged during its implementation. Be-Prox has shown positive effects on decreasing bullying [12]. Additionally, the Early Childhood Friendships Project (ECFP) in the U.S.A. [11,13] was designed as a classroom-based program for young children and consists of a series of content for reducing physical and relational bullying and victimization, and increasing prosocial behaviors. Implementation of this program led to significant decreases in bullying and victimization [13].

These programs have generally focused on young children or their teachers. This is critical for intervention since children and teachers are directly involved in the bullying situation in preschool settings. However, many studies have shown that the family environment and parenting practices influence bullying, thus parents and families should be included in the intervention programs [14,15,16,17,18,19].

### 1.3. Parental Involvement in Stopping Bullying

Parents can play an important role in reducing bullying because parenting style is related to children’s bullying experiences in school, and parents’ attention to school bullying is involved with intervention effectiveness [19]. Lereya et al. conducted a meta-analysis of 70 studies about parenting in relation to peer victimization [17]. They showed that being in the role of the bully and the bully/victim were related to negative parenting, whereas positive parenting practices (e.g., communication with children, warmth, affectionate relationships, parental involvement and support, and supervision) were protective against being a bully or a bully/victim.

Additionally, studies have emphasized that parental involvement is an important factor to maximize the effects of intervention programs [20,21]. The parents’ understanding of bullying may affect whether they respond effectively and appropriately to their child when they disclose victimization [22]. Parental attention and attitude toward bullying, and information or knowledge of intervention can help children cope with bullying effectively [21,23,24,25].

Involving parents in children’s bullying is important, but parents need appropriate information and training because not all adult mediation and coping strategies are helpful for reducing bullying. Fekkes et al. showed that some children who reported their victimization to adults received help, but some children reported that the situations stayed the same or became worse after reporting these incidents to adults [26]. Additionally, some adults told children to seek revenge, inaction, or ignoring the behavior, however, these strategies are not recommended by research [27].

Parents may recognize children’s bullying earlier than the school [21]. They may be aware of their children’s different moods and negative emotions. Young children often did not properly report their victimization experiences to their parents because of their limited language abilities, or desire to continue to play with the aggressor. Parents were aware of their children’s negative feelings, but often dismissed their children were being victimized [28].

Parents’ experiences related to the bullying of their children may impact the strategies they use to intervene. Our previous study indicated that mothers of young children showed different attitudes toward the bullying of their children, depending on whether their children were the aggressor or the victim and whether relations among the mothers were close or not. The mothers of the aggressors or mothers close to the aggressors’ mothers tended to be more generous with regard to the situation whereas the victims’ mothers or mothers close to the victims’ mothers did not [28].

Intervention programs sometimes include parental involvement, however, parents have only been involved to a limited extent [20,29]. Only a few studies on prevention/intervention programs for young children have evaluated their effectiveness, which has been typically measured by school staff or pupils [12,13]. Parents have been rarely involved in evaluating the effects of intervention programs. Thus, it is not known how parents view the effectiveness of bullying prevention/intervention program or policies. Furthermore, the effectiveness of intervention can be seen differently by perspectives. For example, pupils felt that the coping program was not enough to prevent bullying whereas school staff believed that it was adequate [30].

Elucidating parents’ opinions may fill gaps concerning the perception of intervention effectiveness. Specifically, by involving parents in this research, differences between theory and practice could be explained, and we might better understand whether parents’ views differ from those of the researchers or policymakers. This can allow us to understand if some interventions are seen as ineffective by parents. This study focused on the experiences and expectations of bullying interventions among mothers of young children. Furthermore, this study explored the mothers’ perceptions rather than the fathers, since mothers tend to be more deeply engaged in the care of their children than fathers in South Korea [31].

### 1.4. Bullying Interventions in South Korea

The South Korean government has endeavored to reduce school bullying which can be explained by two mainstreams. The first was the establishment of the “*Hakkyo-pokryuk* (school violence) Prevention and Countermeasure Act” and the second was a national project, *Wee*, which is an integrated system for school safety.

In South Korea, all schools have to follow the *Hakkyo-pokryuk* Prevention and Countermeasure Act, which has many articles within it. Among them, two representative articles are described. First, as a proactive policy, schools must provide education about the prevention of school bullying more than once a semester. Second, as a reactive policy, every school must have a committee to prevent and intervene in cases of school bullying (“the *Hakkyo-pokryuk* intervention committee”). This committee may consist of teachers, psychologists, representative parents, medical doctors, or lawyers. Half of the committee members are to be parents of school children who are elected by other parents. If bullying occurs, a committee meeting is held. The children or parents of the children involved in the situation may also attend if required. The committee members discuss the situation and decide upon a solution for the victim and the aggressor. If the problem cannot be solved within the school, the school can seek mediation from local education authorities. Additionally, a child can report his/her victimization directly to the police over the phone (i.e., call 117, a specialized phone number for helping victims of school bullying) or via the Internet (http://www.safe182.go.kr/index.do). In these cases, the police are involved and they often collaborate with school officials to solve the problem. Therefore, *Hakkyo-pokryuk* can be solved by either the school officials or local police, but is more likely to be solved by school officials.

The *Wee* project can be both a proactive and reactive strategy against bullying. It was launched in 2008 by the government and includes a multi-support service network among schools, educational authorities, and local communities, who collaborate to promote student safety and healthy school lives. They support and counsel students on a variety of difficulties. Bullying is one of the main issues of the *Wee* project.

There are three levels of the *Wee* project: the *Wee* class at the school level, *Wee* center at the local educational authority level, and the *Wee* school at the city or province level. The *Wee* project can also be connected to the *Hakkyo-pokryuk* intervention committee or police, for example, bullies can be sent to the *Wee* class, *Wee* center, or *Wee* school after the decision of the committee or police. Currently in South Korea, 55% of schools have a *Wee* class [32], which is located within the school. This is a place for student counseling. The *Wee* class holds regular office hours, and students can visit to talk about their issues. The *Wee* center belongs to the local educational authorities. When schools cannot solve their students’ problems such as bullying, dropping-out, and behavioral or psychological problems, local education authorities intervene. The *Wee* school is a boarding school for high-risk students, which includes psychological treatment, academic education, and special programs for helping adapt for school.

Positive effects of the *Wee* class have been reported. Schools with the *Wee* class had lower bullying and victimization rates compared to those that did not, and this effect was significant among schools that have had the *Wee* class for more than one year [33]. Additionally, schools with a *Wee* class more frequently implemented prevention programs for students when compared to those that did not [34]. However, there is no intervention policy, and no programs for bullying in kindergarten/daycare centers in South Korea. Only elementary-, middle-, and high-school students have been participants in the *Wee* project. Prosocial elements or moral values (i.e., sharing, kindness, cooperation, fairness, etc.) are included in the curriculum in early childhood settings followed by the “Character Education Promotion Act” in South Korea. This includes preventive practices for bullying from a broad perspective, but no specific intervention efforts have addressed bullying among young children.

Lower elementary school students are less likely to be the focus of intervention programs. Moreover, bullying among lower elementary school pupils has rarely been focused on. The South Korean government has investigated the experience of *Hakkyo-pokryuk* for all students from upper elementary school, middle school, and high school students every year, but not among lower elementary school pupils. Thus, it is not known how many children from lower elementary school grades are involved in bullying. Additionally, considering that most young children belong to an educational institution in South Korea [35], there is an urgent need to investigate bullying among young children to establish appropriate interventions.

This study aimed to investigate difficulties, experiences, and expectations of mothers about anti-bullying interventions in early childhood education settings. The aims of this study were to explore: (1) What interventions were used when young children were involved in bullying? (2) What difficulties were experienced while intervening? and (3) What were the mothers’ expectations of these interventions?

## 2. Methods

### 2.1. Participants

Twenty mothers living in Seoul and Gyeonggi Province in South Korea participated in this study. Snowball sampling was used. Mothers who were familiar with the authors and who had experienced the bullying of their children were interviewed and then asked to make introductions to other mothers with similar experiences. Participants were aged 32–47 years, married, and all had 1–3 children aged 1–16 years (at least one child was aged 3–5 years). Most mothers were college or university graduates (one mother was a high-school graduate).

This study conceptualized early childhood from birth to eight years old [36]. In this age range, children in kindergarten, daycare centers, and lower elementary school level were included. These children were exposed to bullying, but their bullying experiences have hardly been investigated in research.

### 2.2. Procedure

Semi-structured interviews were conducted. There were 13 individual interviews (M1, M2, M3, M7, M8, M9, M10, M11, M12, M15, M16, M17, and M18) and three focus group interviews; each group consisted of 2–3 mothers (Focus group 1: M4, M5, M6; Focus group 2: M13, 14; Focus group 3: M19, M20). In many cases, individual interviews were conducted, but depending on the mothers’ schedules, familiarity with the other mothers, and their wishes, a focus group method was also used. These methods provided fruitful information about the mothers’ perceptions of the bullying interventions. The interviewee (second author) did not use specific terms when the interview began. This was intentional because there are several terms used in South Korea corresponding to the term bullying in Western cultures such as *gipdan-ttadolim* (group isolation), *gipdan-goerophim* (group bullying), *hakkyo-pokryuk* (school violence), and *wang-ta* (social exclusion). Each term indicates a slightly different meaning [37]. If the interviewer used a certain term, this may have resulted in biased comments. The terms related to bullying were only used when participants used the term first. Instead, cartoons describing several types of bullying behaviors were shown to the participants, which have been widely used to examine the concept of bullying across cultures [38]. In this study, six cartoons were used, and each cartoon described different types of aggression: physical individual aggression (hitting a smaller person), verbal aggression (saying nasty things), indirect physical aggression (breaking another’s ruler), physical group aggression (several children hitting a child), direct/relational aggression (not allowing someone to play with others), and indirect/relational aggression (spreading a rumor). Next, the participants were asked about their thoughts using the following questions:

- Has your child ever experienced (or have you ever heard about) these behaviors? If so, how did you intervene to stop them?
- What difficulties did you have while intervening in the bullying?
- Have you received any informal or formal support to intervene in the bullying of your children? If so, what support was provided? Who or where did you get help? Were they helpful for you to solve the problem?
- Do you have any expectations or wishes for stopping young children’s bullying?
- What are needed to stop young children’s bullying?
- What types of intervention can be helpful? Who or which institution should provide the intervention?

Interviews took approximately 60–120 min and were held in the participants’ homes or at cafés near their homes. After the interviews, each mother was given a gift card equivalent to 18 U.S Dollars as compensation for their participation.

Some patterns were repeated after ten to eleven mothers were interviewed. However, we conducted more interviews as there were still many patterns that newly appeared. The mothers’ responses or comments were diverse, depending on the bullying experiences of their children. We finished the data collection at the twentieth mother (sixteenth interview) because we recognized that the data had already reached saturation; the interviews did not add any new patterns, and we judged that plentiful information had been obtained.

### 2.3. Ethical Issues

Using an exempt self-determination tool of Chung-Ang University, this study did not require IRB approval. The exemption criteria are that a study should not include children or adults who may not be legally, mentally, or cognitively competent to consent. Additionally, a study should not include the sensitive information of individuals (the Personal Information Protection Act 23 in South Korea). Sensitive information includes data that relates to an individual’s ideology, political views, health, sexual life, or other information that could seriously damage one’s private life. This study did not include any such information.

The purpose and methods of the study were explained to potential participants. The list of interview questions was distributed to the mothers before their participation; therefore, they could decide whether to participate after reading the list. Among the 23 mothers whom the author contacted, three mothers decided not to participate; two had not experienced bullying of their children, and one had scheduling conflicts. After this process, the mothers’ written consent was obtained. Most mothers showed open attitudes and talked actively about their experiences. The participants’ anonymity was respected by using random labels to identify the mothers. For example, M1 was used to indicate Mother 1. In addition, when the children’s names were mentioned, they were only reported as a letter of the alphabet (e.g., A, B, C, and so on).

### 2.4. Qualitative Analysis

All interviews were recorded and transcribed. Nvivo 12 software (QSR International, Victoria, Australia) was used to analyze the data. The grounded theory of Strauss and Corbin [39] was used for the coding process. First, in open coding, the *concepts* of the sentences were extracted. Based on similarities or commonalities among the concepts, the concepts were combined into higher-order code, called *categories*. During the categorizing, the concepts and quotes coded to the concepts were revisited many times, and their suitability was consistently checked. Next, axial coding to compare and contrast among the categories was conducted. Linkages between the categories were made. Some categories were conceptually lower, and included in other categories; in this case, they were classified into subcategories. Finally, in finding overall concepts, a core category was identified. The categories were broken down, queried, and comprised into a higher construct [40]. The most representative and overall concept, *themes* were labelled. Memos and conceptual ideas arising during the course of analysis were written and used through all procedures of the analysis [41].

Through this process, four themes emerged. The authors independently processed the data coding and then held discussions. If discrepancies emerged among the authors, the data and analysis were revisited, and the authors endeavored to find the underlying meaning of the data. To increase the validity and reliability of the analysis process, an independent researcher participated in the process. This individual reviewed whether the mothers’ quotes were coded reliably, and scrutinized the appropriateness of the classification among codes, categories (and subcategories), and themes. Finally, the independent researcher verified whether the analysis represented the phenomena. If disagreements arose, these were further discussed between the researcher and the authors until a consensus was reached.

## 3. Results

The four themes “mothers’ coping strategies”, “problems of interventions”, “expectations of interventions” and “developmentally appropriate interventions for young children” emerged.

### 3.1. Theme 1. Mothers’ Coping Strategies

Mothers used diverse intervention strategies to cope with their children’s bullying/victimization. These were explained by four categories: “direct intervention strategies”, “indirect intervention strategies”, “no response or do not know”, and “deciding to intervene”.

#### 3.1.1. Category 1-1. Direct Intervention Strategies

‘Direct intervention strategies’ means that mothers intervene in the bullying/victimization situation directly by telling a teacher, meeting with the bullies or bullies’ mothers, or bringing their child to the counseling center for psychological treatment. Sometimes, these strategies were used together.

“I met the aggressor’s mom, I phoned her and asked to meet. I told her that I can’t sleep whenever I see my child’s wound.” (M14).

“It was really severe and frequent. I told a teacher and asked not to be in the same class with my girl and that child (aggressor).” (M20).

“One girl told *F*, ‘You weren’t in my class, you have come from the other kindergarten, you are not my friend!’ So, *F* cried every day and didn’t want to go to kindergarten, I went to kindergarten with *F* and stayed at *F*’s side every day.” (M6).

“I brought *T* to the counseling center which was run by an NGO, *T* received counseling for three times.” (M20).

#### 3.1.2. Category 1-2. Indirect Intervention Strategies

Indirect intervention strategies refer to intervening in the situation through the children. This was the most frequent response reported by mothers. Mothers used active and passive indirect strategies. The subcategories “active-indirect strategies” and “passive-indirect strategies” explained the category.

##### Subcategory 1-2-1. Active-Indirect Strategies

Mothers explained that children have to do something to reduce the bullying, so they taught their children what to do, or they learned coping strategies to teach their children.

Generally, mothers used positive strategies such as teaching social and emotional skills to their children to react appropriately; these skills related to solving peer-conflict positively, maintaining relationships, increasing empathy, and communication skills.

“I said to *B,* ‘Put yourself in the other child’s shoes’…then *B* told me, ‘He (she) would feel bad…’ then *B* seems to think that she should not do it next time.” (M2).

“I told *P,* ‘You approach slowly and say something to her, rather than just look at her without words’.” (M16).

Additionally, some mothers wished their children would not exaggerate and tell just as it is.

“I didn’t imagine at all that my child did do that, I believed ‘My girl is really innocent, naive, and a real child’… But I found out that she made up stories when she was in a disadvantageous position... I saw it, so I told her to say as it is…If a teacher asks you, just tell only what you see.” (M19).

Mothers noted that adults needed to support children to solve problems by themselves rather than directly intervening in their children’s relationship problems.

“Adults’ intervening should be wise, if we want to intervene, indirect coaching is better. If we do directly intervene in the relationships among children, children’s conflict moves to conflicts among parents of the children.” (M11).

In some cases, mothers instructed their children to react in the same way to the aggressors (i.e., revenge).

“When *E* was hit for the first time, I said, ‘You should hit the child next time. You hit the child as much as you were pained’… *E* hit the other child, I asked ‘How did you feel?’ *E* said, ‘It wasn’t good’.” (M5).

Additionally, mothers learned from other mothers or obtained information through the Internet concerning how to respond to their child’s victimization.

“I searched books first, psychological books in the library. I lack parenting skills. I studied how to raise a boy, and watched TV programs related to parenting…I searched information through the Internet related to ‘a bully’…” (M18).

##### Subcategory 1-2-2. Passive indirect strategies

Sometimes mothers told their children to ignore or avoid the aggressor and play with other children. 

“Make other friends first, and get along with [that] child later.” (M2).

#### 3.1.3. Category 1-3. No Response or Do Not Know

Some mothers trusted the mother of the other child or did not know how to do. Sometimes they just put up with it.

“I just thought, ‘It is children’s matter’…I trusted her [the aggressor’s mother].” (M8).

“I didn’t know what to do. I didn’t have wisdom, or experiences about this. I didn’t know the situation enough. I just have my child’s words…I didn’t know what will happen, what to do... I felt helpless.” (M16).

“I don’t have any certain answer. I can’t say to *S,* ‘You hit as the child did to you’ nor ‘Just stay and do not hit back’…so I told *S,* ‘Ask help from an adult’…but the teacher says, ‘Do not tattle’…There is nothing *S* can do…so I don’t have any words for *S*…I am helpless... Do I have to say to *S,* ‘Run away?’ it is so sad, very sad…I really do not know how to tell *S.*” (M19).

“My boy was hit on his face really seriously in kindergarten. I really wanted to rush to the child and do something to him/her. I have never hit my child. I was sleepless for a week… but I thought something more serious like an accident might happen while he grows up so I did not express my negative feelings to him.” (M14).

These reactions are important because the mothers’ lack of responses did not mean that they did not care about the situation. Rather, it can be interpreted as an “inability” to react although they wanted to act against the victimization of their children. Mothers did not react because they thought that their child may be agitated or made more anxious by their emotional responses. The mothers’ rational responses may be appropriate; however, they could have acted by teaching children or seeking help from others.

#### 3.1.4. Category 1-4. Deciding to Intervene

This category describes how mothers used the diverse coping strategies explained above. The subcategories are “understanding the situation correctly” and “discrepancy between mother and father on intervention strategies”.

##### Subcategory 1-4-1. Understanding the Situation Correctly

They emphasized that understanding the situation correctly was most critical and the type of strategy differed in each bullying situation.

Understanding the bullying situation correctly was frequently mentioned; this was important for the mothers before acting or using an intervention.

“I phoned the aggressor’s mom, and told the story…later, I found that she heard a completely different story from her son. If I didn’t know the truth, I would have asked the *Hakkyo-pokryuk* intervention committee.” (M19).

Mothers used various strategies such as direct and indirect intervention strategies depending on the bullying situation, and its severity. Additionally, several interventions could be used together.

“At first, I listen to my child’s words, and lead my child to understand the other child’s mind. ‘Why the child did it to you?’ If it doesn’t get resolved, adults need to help such as teachers, and the other child’s mom. Adults need to interfere in the issue…If this doesn’t work, I will leave the kindergarten or school.” (M17).

##### Subcategory 1-4-2. Discrepancy between Mother and Father on Intervention Strategies

Discrepancies in the desired means of intervention arose between mothers and fathers. The discrepancies influenced mothers to decide on appropriate intervention strategies. Some mothers complained that their husbands did not respond thoughtfully on this matter.

“Fathers often do not think this as serious. They think ‘Children are grown up like that.’ Mothers are upset. I am really agonized when my child comes home with cry.” (M3).

In contrast, some mothers explained the difficulty of dealing with the strong reactions of their husband, so sometimes they told only part of the stories to their husbands because they were afraid that the situation would become much more severe, or difficult to manage.

“I did not tell my husband about the details. Men do not stay silent as do women. They protest vigorously, they check whether the situation is administrative or lawful. That is why teachers are cautious to tell fathers. Men bear in mind and react strongly in a sudden. Mothers care of relationships, but fathers do not…I am anxious that he is gone too far.” (M10).

M10 mentioned anxiety over the reaction of her husband, but she did not seem to be negative to the active reaction of her husband. The fathers’ active reaction seemed to make the mothers relieved, even if it was sometimes an over-reaction. In contrast, the fathers’ indifference was problematic for the mothers to manage the matter.

### 3.2. Theme 2. Problems of Interventions

The theme “problems of interventions” included problems and difficulties with the current intervention policies and programs. Problems were related to the counseling center and school/preschools such as kindergarten, daycare centers, or both. The two categories “counseling center/psychiatry-related” and “school-related” explained this theme.

#### 3.2.1. Category 2-1. Counseling Center/Psychiatry-Related

The mothers used counseling centers, which can be run by the local authorities or government, or run by professionals in child development centers, and visited child-psychiatric clinics. Regarding the counseling centers and psychiatrists, the mothers complained about the difficulty of access. Furthermore, follow-up treatment was not conducted at those centers therefore the effectiveness was only temporary. The four subcategories of “lack of counseling centers”, “physical and psychological distance”, “high costs”, and “temporary effect” explained this category.

##### Subcategory 2-1-1. Lack of Counseling Centers

“There are few places to get support. There is little information about counseling centers.” (M2).

##### Subcategory 2-1-2. Physical and Psychological Distance

Physical distance to the center made it difficult for the mothers to visit.

“It is far from my home, I hesitated whether I need to bring my child to that far, I asked myself ‘Is my child that serious to go that far?” (M1).

Due to the psychological distance, mothers expected the problem to be solved within the school/kindergarten.

“The Center is a third party, they don’t know our children as the kindergarten does, it feels disconnected from us.” (M11).

##### Subcategory 2-1-3. High Costs

High costs were a problem that prevented some mothers from visiting psychiatric clinics. Some mothers hesitated in using these services because of the costs, but some mothers were willing to pay for it as long as the psychology or behavior of their children could be improved:

“I heard that it was 100,000 won [around 89 U.S dollars] per psychiatric counseling session. A friend of mine [aggressor’s mom] said ‘It is not a waste if my boy could grow up righteously. It is better than being a bad person later.’” (M9).

##### Subcategory 2-1-4. Temporary Effect

Mothers reported on the effectiveness of counseling or psychiatric treatment. However, the effectiveness was seen as temporary.

“If the treatment of a psychiatrist is effective, the expensive fee is fine, but the effects don’t last long... During treatment, the child looks fine, but as time goes by, the effects are fizzled out.” (M2)

#### 3.2.2. Category 2-2. School-Related

This category explains the mothers’ difficulty or dissatisfaction related to school or preschool intervention. The four subcategories of “lack of knowledge”, “stigma”, “superficiality”, and “teachers’ stress” explained the category.

##### Subcategory 2-2-1. Lack of Knowledge

The lack of knowledge about bullying was frequently explained in terms of class teachers and the organization of the committee members of *Hakkyo-pokryuk*. *Wee* class teachers (who were involved only in counseling pupils at the schools) were professional counsellors; however class teachers were not experts. Teachers in kindergarten or daycare centers were not aware of *Hakkyo-pokryuk* or bullying. Mothers pointed out the problems with members of the committee. Most schools have committee members who are parents and only sometimes have professionals such as lawyers or doctors. This resulted in lack of knowledge of the *Hakkyo-pokryuk* intervention committee.

“The *Hakkyo-pokryuk* intervention committee is not for solving children’s problems in school, rather it looks like a place for expression of parents’ own opinions.” (M16).

“Often the committee members are parents. Thus, some committee members who are parents may be biased to judge the bullying situation.” (M14).

Additionally, kindergarten teachers’ unskillful intervening techniques for bullying made the situation worse.

“In the kindergarten there was a child who is developmentally advanced. He bullied other children, so teachers suggested sending the child to upper class where one-year older children belonged. The suggestion was formally shown without parental admission. Mothers did not agree of it. Teachers suggested the solution clumsily.” (M9).

The untrained teachers and unprofessional members of the *Hakkyo-pokryuk* intervention committee led to many problems and caused parents’ emotional distress rather than solving the conflict reasonably.

##### Subcategory 2-2-2. Stigma

Lack of knowledge about bullying among the school/preschool staff can generate stigma among children who are “trouble-makers”. The lack of management of the intervention system may have contributed to the problem.

“*L*’s cousin said that *Wee class* is a kind of threat which is used by teacher. ‘If you keep doing like that, you may be sent to *Wee class’*…The teacher of my child says, ‘You can go *Wee class* if anyone needs to, you can talk whatever you want and play there’…but teachers in other schools say, ‘*Wee class* children! Come here and follow me’, they bring the children to *Wee class.*” (M20).

“If *W* and *Y* fights, the committee is held, even if they were 1st graders, but the situation is spread even to fourth and fifth graders, their names, class, etc.… Then, the aggressor would be fine because other children will not bother or tease him/her. Victim is seen as pitiful to the children. The aggressor is stigmatized as a bad child among mothers in the committee.” (M14).

##### Subcategory 2-2-3. Superficiality

Mothers reported that the intervention system was superficial because it did not correct bad behavior nor did it address the children’s motive for bullying.

“I don’t think it is a good idea to make children apologize to each other in public, I have never seen them do it sincerely.” (M17).

“A policeman comes up in the *Hakkyo-pokryuk* intervention committee and greets mothers, but I do not trust him/her at all. It is like a ‘performance’, to show someone. I don’t want it. Rather, they should approach children friendly ‘Hey, I am here, whenever you feel afraid, call me’…” (M14).

“It does not grasp problems, each child (victim or aggressor) has his/her inside. It does not analyze nor lead children to reconciliation. It neither teach nor guide children. Rather it does ‘this is your fault,’ ‘this is teacher’s fault.’ Children’s victimization is problem, but also parents who do behave like that (in the committee) are problem.” (M16).

Mothers distrusted the solutions such as forcing apologies between the children or just judging the children’s fault without being aware of their minds. It is doubtful whether adults have a strong will to solve children’s problems. Alternatively, adults in the committee might not know appropriate, efficient, and helpful ways to assist the children. Particularly, the procedure of the committee was problematic, and this may come from a lack of knowledge about bullying among the committee members.

##### Subcategory 2-2-4. Teachers’ Stress

Although mothers complained of the teachers’ lack of knowledge to cope with bullying, they also appreciated the level of stress that teachers experience may distract their attention from bullying and interrupt the development of coping skills for bullying. Teachers experienced a lack of support in the workplace as well as stress due to their interactions with parents.

“Teachers got hurt from parents. I have heard that teachers cry. Teachers in daycare centers do not have break time, they do not have annual leave, they cannot manage the work related to bank because banks close at 4 pm. If they confront mothers on the way to the bank, that can be gossip among mothers. So I have heard that they disguised when they need to go out.” (M9).

“My friend is a school teacher. When the committee of *hakkyo-pokryuk* was held, the parents assaulted her, she was so stressful that she got herpes zoster. The day after, the parents apologized her. What is that! Humiliating a teacher in front of other parents and saying sorry next day.” (M16).

### 3.3. Theme 3. Expectations of Intervention

The mothers’ expectations of interventions comprised three levels: the categories “family level”, “school level”, and “local authorities/government level” explained the mothers’ expectations. In addition, “pessimistic for prevention/intervention” was indicated.

#### 3.3.1. Category 3-1. Family Level

Mothers emphasized an affectionate home environment and its role in children’s character development. Four subcategories of “parental responsibility”, “affection and attention”, “character and moral education”, and “self-reliance” explained the category.

##### Subcategory 3-1-1. Parental Responsibility

Mothers should be careful about the behavior of their children; and should not overreact about their children’s victimization.

“Parents are better not to be sensitive. If they are, children would keep reflecting it. I expect my child is not a sensitive soul.” (M17).

Early discovery of a child’s problem is the responsibility of their parents.

“Mothers should find a child’s problem such as anxiety or violent characteristics as early as possible, she may bring the child to a psychological therapist.” (M4).

##### Subcategory 3-1-2. Affection and Attention

Parents’ affection and attention concerning children’s daily lives are fundamental.

“Parents must care for children. They have to pay attention to their children, always…They have to ask themselves ‘Is my child doing well in school?’… Mothers’ role is most important until a child grows up as a member of society. I am keen to my child and support him/her.” (M12).

However, one mother reported that “ignorance is bliss”. She was afraid of being neurotic if she knew all of the details about the story. M9 seemed to avoid the situation and was distressed about her child being bullied.

“Sometimes, if mothers do not know, children can have opportunity to make relationships with others, then they can solve the problem by themselves. If I hear the story from other mothers, I am inquisitive and ask my child to check, ‘How was that child today?’… If I know something, which can make me anxious. I hope I do not know and my child does well.” (M9).

##### Subcategory 3-1-3. Character and Moral Education

Mothers emphasized character and moral education at home. This can be achieved by increasing the ability of children to empathize. Conflicts among young children occur over very trivial things, therefore adults need to teach children the means to obtain what they want without damaging relationships or others’ emotions.

“Actually, in kindergartens, children want to have toys which others have. Then they can fight… I says to *P,* ‘A friend may want the toy you want… you should not take out what others have. You should get along with others. You have to acknowledge what others want’ … The other day, *P* pushed another child… I keep saying ‘That is wrong and bad behavior’ until *P* understands.” (M16)

Additionally, character education should be specific rather than the rote learning of ‘good things’.

“Preventive strategies are too wide to inform them one by one… They should go with character education… Just simply saying, ‘You should not play with *X*’ is not specific because my child can be both aggressor or victim. So, character education is fundamental to prevent these behaviors... I teach my child proper behaviors.” (M12).

##### Subcategory 3-1-4. Self-Reliance

Mothers highlighted children’s self-reliance; they must learn coping skills against bullying, because it could happen throughout their lives.

“I will teach *G* to have autonomy, and self-reliance. *G* should express himself/herself, like ‘You must not do that to me’… Mothers cannot solve everything though they can help… A child should stand up to the aggressor.” (M7).

#### 3.3.2. Category 3-2. School Level

At the school level, teachers were the most important in terms of preventing bullying. In addition, a change of curriculum was suggested. The category was explained in terms of the five subcategories: “teachers’ role”, “increasing number of teachers, *Wee* class teachers, and counsellors”, “collaboration among mothers, teachers, and psychiatrists/counsellors”, “providing more opportunity for plays and physical activities”, and “social education programs”.

##### Subcategory 3-2-1. Teachers’ Role

There were many comments about the importance of teachers. Mothers mostly emphasized that teachers were very influential, especially for young children.

“Teachers’ intervening is most effective because parents’ and teachers’ words are influential for young children.” (M17).

“The best solution among several strategies I tried was to ask the teacher. It was definite and the firmest solution because the teacher acknowledged the situation very accurately.” (M16).

##### Subcategory 3-2-2. Increasing Number of Teachers, Wee Class Teachers, and Counsellors

Mothers understood the teachers’ difficulty in caring for many children in one class and they felt children would have more opportunities for counseling if the teacher–child ratio decreased.

“More teachers are needed… If the ratio of teacher-child is low, teachers would take care of children better.” (M15).

“Teacher-Assistants are necessary. If a child wants to go to the nursing room, there needs to be someone who brings the child there. Also, the TA could check whether a child really goes to the nursing room, or goes to play around.” (M19).

“One counselor in one school is really lacking, it doesn’t make sense.” (M10).

The number of professionals should be increased within educational institutions to make it easier to take care of children and supervise bullying.

##### Subcategory 3-2-3. Collaboration among Mothers, Teachers, and Psychologists/Counsellors

Mothers, teachers, psychiatrists/counsellors can support each other as they each have a unique role:

“If a teacher mediates well between mothers, the victim’s mother could understand the situation, but if the teacher leaves mothers to meet and talk by themselves, the mothers couldn’t solve it. They are just on their children’s sides. They may be emotional… Teachers could be in between aggressor’s and victim’s mothers. Teachers balance the situation, but mothers believe their children’s words.” (M20).

“A school nurse is dispatched in every school, similarly, psychological counselors may join in the incident and do what kindergarten teachers cannot do. They can work together.” (M8).

##### Subcategory 3-2-4. Providing More Opportunity for Plays and Physical Activities

Mothers expected a diminishing number of academic classes and an increase in more free play with peers, which can help with the children’s social and emotional development.

“I want group play more, not individual play in kindergarten.” (M9).

“I wish elementary school curriculum focused on morality or diversity rather than on study. *G* learns English three times per week in the daycare center, but I think playing is better than that… playing, running…” (M7).

##### Subcategory 3-2-5. Social Education Programs

A social education program needs to be accentuated from early childhood.

“I think making relationships is necessary for children. They have to develop their competence for it. School needs to teach this. People all have different characteristics, so children should learn about that and respect it.” (M5).

“Children have to know the possible consequences which can be caused by certain behavior. The program related to *Hakkyo-pokryuk* show this. Why not kindergarten?” (M12).

Most mothers suggested various ways to resolve conflicts through social education rather than punishing strategies. There was one mother who mentioned her experience that the intervention strategies could differ among mothers.

“Some mothers insisted punishment whereas some mothers said that it was too harsh. Some react strongly, other mothers step back. I have heard that there were more mothers who wanted to leave the issue to the school.” (M2).

#### 3.3.3. Category 3-3. Local Authorities and Government Level

Mothers’ expectations of local authorities or the government covered a wide range of interventions. This was explained by the three subcategories of “establishing counseling places”, “improving the sense of community”, and “systematic and sustainable public interventions”.

##### Subcategory 3-3-1. Establishing Counseling Places

Mothers expected places where they could talk about their difficulties related to their children within the preschool or their local areas.

“If a child is victimized, centers which manage parents’ stress and difficulty would be helpful. They can tell us other cases, then we may feel, ‘Oh this is not only my issue, other people also have similar difficulty… Then sometimes we can find a solution by ourselves.” (M10).

##### Subcategory 3-3-2. Improving the Sense of Community

Parents needed to consider other families’ perspectives. Parents’ selfishness made the situation worse.

“Parents generally think ‘My child does well, why did the other child do that?’ This may elicit an aggressive attitude toward others. My child can be wrong. It is better not to think only from my child’s perspective.” (M16).

Parents needed to communicate and understand each other during parent meetings.

“Mothers are looking for a breakthrough. It is not for just a simple chat, it should be more educational to see things positively and change their thinking… That kind of meeting is needed.” (M18).

Peaceful and relaxing local places would be helpful for families’ happiness or harmony; families can refresh and relieve their stress by visiting places such as parks, gardens, and playgrounds, which may provide opportunities to communicate between family members and increase a sense of belonging to the local community.

“A new playground in a forest near my block was built, and family gardening was open. We played there many times. Providing something to local people is better than receiving a superficial message like ‘report to police’ from the local office. I and husband shared our thoughts with our children.” (M9).

##### Subcategory 3-3-3. Systematic and Sustainable Public Interventions

Mothers required that collaboration among home, school, and government levels of care should be systematized. A coping manual for bullying was necessary.

“I think there must be a manual in educational institution such as stage 1, stage 2, etc. So, teachers can follow the manual if an accident happens. For example, stage 1. Calling parents, stage 2. something, and stage 3…” (M4).

All interventions should be consistent and sustainable.

“I guess there must be education related to this in public institutions. Daycare centers can search and use those educational programs, even once a semester or once a month. Fire alarm safety training is regularly done though it hardly happens actually. But, there are very few social education things like emotional education, and peer relationships, though these things happen in everyday life.” (M11).

“I expect that teachers might tell children about this matter regularly.” (M20).

“From early childhood including low grade in elementary school we should have our eyes on *goerophim*… Caring and educating children only at home is not enough, schools and home have to counter this issue together… I hope the local community helps much more …I wish there were many home related activities such as visiting a home regularly, family connected program, or encouraging fathers’ participation on parenting… These things finally decrease *goerophim* because the family becomes happy.” (M3).

#### 3.3.4. Category 3-4. Pessimistic for Prevention/Intervention

Some mothers showed pessimistic attitudes toward the intervention or prevention efforts. They thought that bullying could happen anywhere, and that bullies always exist unless all families are affective and all parents provide warm parenting, which does not actually exist. They felt that institutions could not solve the fundamental causes of bullying.

“If my child is victimized, it cannot be solved by the support from kindergarten or school... I would invite the aggressor, be nice him/her, ask ‘what do you like?’ and then at later ‘how do you think about my child?’ An opportunity is needed to solve the problem at first stage but these cannot be done by teachers or school staff.” (M8).

“Is prevention possible? I think it is not, because home atmosphere or parenting style may affect children’s behavior in schools. So, to prevent bullying, changing family or parents is essential but this is almost impossible… These children exist wherever, and whenever. However, interventions to stop bullying are necessary. Counseling centers or supporting places should be located within the institution.” (M1).

The mothers were doubtful and pessimistic about preventing bullying. However, they had their own ideas about how to solve the problem such as inviting the aggressor or establishing support centers. M8 was opposed to support from institutions whereas M1 believed it was necessary to stop bullying.

### 3.4. Theme 4. Developmentally Appropriate Intervention for Young Children

Mothers strongly suggested the need for a program that included contents of young children’s social-emotional, and psychological developmental status. These were described by the three categories “using various resources”, “consideration of concepts and terms”, and ‘ways of expression and language ability”.

#### 3.4.1. Category 4-1. Using Various Resources: Real Objects, Materials, Videos, Puppets, Storytelling

Mothers explained that social skills were essential for children. To increase the children’s social skills, various materials were suggested such as videos, puppets, and storytelling using hypothetical situations.

“I am not sure whether the videos exist…those show peer relationships among young children… Social relationships programs are necessary… The materials could be used both at home or in kindergarten. Teachers or mothers are able to educate with the materials- fairy tales, or video, dramas…” (M11).

“Use like a role play, or storytelling, puppets. Children could tell their experiences related to the story which the teacher tells.” (M20).

Making and showing educational videos for young children was most commonly mentioned by mothers. The videos should show situations that children may experience in their daily lives, thus explaining what bullying is and how to cope with it.

“The program should specify children’s peer conflict situation. Just saying ‘hitting is bad’ is not enough… Using examples of the situation would be helpful.” (M5).

#### 3.4.2. Category 4-2. Consideration of Concepts and Terms

As the young children’s concept of *Hakkyo-pokryuk* was unclear, it was unsuitable for them to be educated using the term.

“I have heard that one child reported ‘He/she was hit’ to the police. He/she could have told this to his/her teacher. He/she did not consider its severity and just directly phoned to police, the problem became very big.” (M6).

“Young children do not know what *Hakkyo-pokryuk* is, but, they use the term even if a friend teases them a little. So, the term is regarded as not serious to them.” (M19).

“The first grade in elementary school looks like an extension of kindergarten. The first graders are very similar to kindergarteners… The term *Hakkyo-pokryuk* has strong nuance to young children.” (M20).

Acknowledging the severity of aggressive behavior is an important issue because it affects judging whether it is bullying, and whether it needs to be reported to the police. Young children seem to have difficulty in distinguishing *Hakkyo-pokryuk* from other aggressive behavior such as fights.

This is why various materials should be used to help young children in their understanding of bullying.

#### 3.4.3. Category 4-3. Ways of Expression and Language Ability

As young children are not fully able to express their experiences with language, other ways to express their emotions and experiences should be considered.

“Children in lower grades in elementary school have difficulty with counseling because they are clumsy when expressing themselves. Art therapy or psychological therapy would be better for them, once or twice a month. A program that lets us know children’s inside may be helpful. There is nothing.” (M2).

As both a proactive and reactive strategy, understanding children’s psychology is important, especially among young children whose language is restricted. This is also why the *Hakkyo-pokryuk* intervention committee may be not appropriate for lower grades in elementary school pupils as the committee sometimes requires the children to explain the situation.

## 4. Discussion

This study investigated mothers’ experiences and expectations concerning bullying interventions among young children. Mothers used their own coping strategies, which had not been learned in public or systematically. They intervened in their children’s bullying either directly or indirectly. The coping strategies mothers used in order to intervene in their children’s bullying were sometimes effective and sometimes ineffective. The most problematic issue was having ‘no information’ about bullying and intervention programs for young children. At the elementary school level, mothers were anxious regarding less specialized teachers on *Hakkyo-pokryuk*. This may lead to children being stigmatized and may mistrust intervention systems. In kindergarten/daycare centers, the lack of places for counseling and lack of knowledge about bullying amongst the teachers were the main problems. Mothers showed their expectations with regard to intervention for young children from multi-contexts (i.e., family, school, and local/governmental level). These results provide many academic and political implications for the intervention of bullying among children.

### 4.1. Mothers’ Intervention Strategies

The mothers predominantly used indirect intervention strategies such as teaching children social skills, assertiveness, or appropriate behavior. They sometimes used direct strategies by asking teachers for help or contacting the aggressor’s mother. These strategies could be used together depending on the severity or repetition of the situation.

Mothers felt responsible for solving their children’s bullying/victimization problems, but they often felt helpless because they felt unprepared to solve these problems. Mothers who did not respond to their children’s bullying did not mean to convey that they did not care; rather, these mothers did not know what to do. This implies that institutional interventions should actively support mothers. Moreover, the mothers’ strategies were made at a personal level, and not at an institutional or public level. There are no public manuals to inform mothers, and few places for mothers to visit for support. As Axford et al. [19] explained, successful intervention at the individual level is difficult to achieve and is more likely to be reactive than proactive.

### 4.2. Disappointment with the Intervention

Mothers pointed out the lack of a system or facilities that could offer them help. At the preschool level, this was more problematic because the mothers of children in kindergarten/daycare centers relied on teachers or counseling centers/psychiatric clinics. There was no formal network or system to solve these problems at the preschool level. Counseling centers or psychiatric clinics were not easy to access due to high costs, psychological distance as well as physical distance. Additionally, mothers felt that the intervention effects were temporary. The temporary effects may have resulted because the intervention strategies used by the mothers were disconnected; discussing the problem with the teacher and visiting counseling centers operated independently, although there is a need for them to work together to boost effectiveness.

Lack of knowledge about bullying among teachers was consistently noted as a main problem with the interventions. Kindergarten/daycare centers tried to mediate bullying among children, but the teachers were typically unaware of the instructions and had not been educated on how to teach children appropriate coping strategies when bullying occurred. At the elementary school level, a lack of knowledge about bullying among teachers was part of the incompetent management of the *Hakkyo-pokryuk* intervention committee and *Wee* class. Moreover, many parents serving on the committee did not have specialized knowledge about bullying, therefore their decisions were not considered credible to other mothers. The incompetence of teachers or school staff may lead to the stigmatization of children involved in the incidents, which may negatively affect the children’s willingness to participate in the intervention programs.

Developmental consideration for younger groups was shown. Lower grade students in elementary school were often excluded from the intervention program. Their bullying behavior was sometimes managed by teachers or parents and was sometimes dealt with by the *Hakkyo-pokryuk* intervention committee. However, the committee does not seem to be appropriate for young children since it was designed for older children.

### 4.3. Mothers’ Expectations for Intervention

Mothers expressed their many expectations related to the intervention policy and programs for bullying. These were explained in terms of the family, school, and at the local authorities/governmental level, which is consistent with developmental ecological perspectives [16]. In bullying, individual and contextual factors are interrelated from a proximal system (children’s parents, friends, etc.) to a broader context such as cultural norms and beliefs. Therefore, for a prevention program, multi-level contextual factors should be considered [16,42,43].

According to the mothers we interviewed, the most fundamental prevention method was within the family, particularly that of parental affection and character education, which has been stressed in previous studies [17]. Affectionate parenting styles allow children to talk freely to their parents about their difficulties, which provides them with chances to learn coping skills to prevent further victimization [42,44]. Additionally, regular communication between children, teachers, and parents is important to prevent and intervene in bullying as 25% of bullied children do not tell adults, which may exacerbate victimization [26].

For young children, self-reliance was emphasized as they may confront similar or more severe bullying situations as they grow older. This is similar to teaching assertiveness to young children as an important strategy [45,46]. Mothers expected that the problem would be solved within the preschool as the preschool staff know their children well. This reflects that training for teachers about bullying is necessary, which has been emphasized in many studies [47,48].

The most reliable intervention mentioned by the mothers was the teachers’ competence. Mothers seemed to rely on the teachers’ competence to solve the problem as a mediator. For mothers, teachers are physically and psychologically closer to their children than counselors or psychiatrists. Training teachers in preschool is effective in sustaining the effectiveness of the intervention because teachers consistently care for children at least for one year whereas counsellors are connected only for a short time.

Therefore, a systematic manual should be established, which means developing a protocol specifying the process or stages about how teachers or parents can respond when bullying takes place. For example, at Stage 1, teachers should pay attention to the situation and the children’s words. In Stage 2, teachers should decide what kind of intervention is appropriate for the situation. For example, the model for conflict mediation suggested by Kostelnik et al. [49] might be useful (i.e., initiate the mediation process → clarify each child’s perspective → sum up, generate alternatives → agree on solution → reinforce the problem-solving process → follow through). Teachers should also determine whether the children should be separated (if they are harming each other), and whether parents should be told. In Stage 3, if these efforts fail, teachers can be connected to specialized institutions or counseling centers. For this, a local network between schools/preschools and counseling centers/child psychiatrists needs to be created, and access must be easy and welcoming for mothers.

More professionals should be included in the *Hakkyo-pokryuk* committee, and the government should strictly control these criteria. Alternatively, a third party such as the local authorities should intervene when necessary. This may lead mothers to view interventions as more authoritative and effective. Smith also suggested considering the balance between school policy and action centrally [48] (p. 421).

The superficiality of the intervention program was pointed out. To prevent further bullying, it is fundamental to find out the reason for the bullying behavior and the course of the incident rather than judging fault. Furthermore, bullying and victimization are often related among young children [3,4,50]. Similarly, many mothers in this study viewed the young aggressor as a possible victim of negative parenting or a deprived home environment. One mother in this study reported her experience about some mothers insisting on punishing the aggressor. However, most mothers of young children in this study were less likely to blame children involved in the bullying. Rather, they considered that the parents of aggressive children should be changed and suggested that social support was needed for good parenting skills and a positive family atmosphere to the change home environment.

This corresponds to the theory of the restorative approach. The basic assumption of the restorative approach is to resolve conflict, compensate hurt, and rebuild good relationships rather than blaming bullies. Bullies acknowledge the consequences of their behavior, empathize with the victim’s emotions, and compensate for the victim’s pain [51]. Intervention programs using a restorative approach have been shown to be effective [52].

One mother said, “Children are still young. Deciding who are bullies and victims is adults’ judgment. Asking them to reenact the situation is not appropriate. It is caused by adults’ emotion (who are upset for the situation). Children are not that mature yet.” (M14). This implies that intervention policies should be based on the children’s perspectives. Adults develop intervention systems. They wish to clarify and solve the incident through many formal processes and system; how distinguish aggressor from victim, and how make them take responsibility of their behavior, which is retribution justice [51,53]. It is important to prevent bullying by viewing bullying from the children’s perspective, which is very important, especially for younger children.

Few studies have suggested a restorative approach for young children’s bullying, however, it is not clear how it could be applied in young children’s bullying intervention. For example, circle time could be considered where a teacher can discuss the issue to elicit a solution with a whole class.

Social and moral education were emphasized. This is not new as it has been consistently and widely included in bullying prevention programs [54]. In particular, the home is a vital place for shaping one’s character, personality, and school is an optimal place for practicing social skills with diverse individuals. This implies that the cooperation of families and schools are necessary, therefore, intervention program for families should be developed.

In South Korea, social and character education have predominantly been achieved through informal curriculum (i.e., latent curriculum) in schools/preschools rather than through special courses or academic subjects. However, social and moral education need to be arranged in formal education so that they can be emphasized consistently on a regular basis. Through social education at school, children may practice and develop their social skills with many peers. Since young children’s language abilities, emotional competence, and social skills are still developing, schools or preschools need to teach them to develop and practice their skills and abilities, thus young children are able to solve their conflicts more peacefully and in socially acceptable ways. For example, SEL (Social and Emotional Learning) programs are widely used to develop emotional ability and decrease behavioral problems [55]. SEL has been developed as a school-based program from kindergarten to high school and it has been shown to be effective for students’ social and emotional well-being [54]. The effectiveness of SEL has been shown for reducing aggression though the program is not directly related to bullying behavior [56,57].

Increasing the number of counseling centers for parents and children is required. There are no specific programs for bullying prevention/intervention. At the preschool level, an establishment corresponding to the *Wee* class is needed, which could be located within the preschool or within the larger community.

There has been much debate on the effectiveness of “bullying-focused” solutions, or more general solutions based on relationships and school climate and improving ‘conviencia’ (in Spanish, implies respect and co-existence) [51] (p. 327). Current intervention systems in South Korea (i.e., the *Hakkyo-pokryuk* intervention committee, *Wee* class) are closer to a “bullying-focused” solution. Moreover, these do not seem to function effectively unless the school staff are trained. Social and character education needs to be formalized to maintain its sustainability, whereas the procedure in the *Hakkyo-pokryuk* intervention committee is superficial and lacks substantive content, so the proactive system should be fortified. Young children’s developmental characteristics do not fit into the bullying-focused system, and they have difficulties in controlling their desires and behaviors without appropriate social and moral knowledge and guidance. This study indicates that the focus of intervention for young children should be shifted from a reactive to proactive means.

### 4.4. Suggestions for Intervention for Young Children

(1) Family-based intervention program

Family-based intervention programs are crucial. Most mothers have difficulties in coping with bullying situations. The program should include general proactive to specific reactive solutions; knowledge about effective parenting styles, character/moral education, and coping manuals for bullying.

Generally in South Korea, schools/preschools regularly distribute a newsletter to parents, which covers a wide range of issues in schools/preschools; however, this is not sufficient for preventing bullying. More active programs or school policy are required (e.g., parent meetings and education about bullying). The FSFF (Friendly Schools Friendly Families) could be a good example [58] as it focuses on parents’ attitudes, knowledge, and their self-efficacy to help children with bullying. Its effectiveness was maximized when high-intensity intervention was made at the whole-school level and family level [59]. Increasing parents’ awareness of bullying was performed through diverse ways such as newsletters, booklets, songs, referral information, parent workshops, parent–child communication sheets, and class–home activities [59]. These activities may be useful for developing bullying interventions for parents.

Additionally, Butler and Platt developed a structured family therapy model to help by incorporating the families, teachers, and therapists. School counselors and teachers are part of the family therapy, and therapists are connected to the school directly during treatment. In this family based intervention program, the collaboration between professionals and parents is strongly emphasized [29]. Local authorities or government could help to establish the network between professionals, teachers, and parents, and they could also offer these programs to the local communities.

(2) Establishing intervention policy and program for young children

Increasing the teachers’ specialization is an urgent issue as they require professional knowledge regarding bullying. Policies regarding the bullying of young children as well as for teachers are required. In South Korea, pre-service teachers in university or college must complete a course about school bullying prevention and intervention in university or college. Pre-service teachers in early childhood also take this course, but they learn about bullying at higher levels and do not learn about bullying in preschool. Thus, anti-bullying training in early childhood education should be separately established. Furthermore, for in-service teachers in preschools, education about bullying should be implemented on a regular basis.

Intervention programs should distinguish the lower grade students from those in the upper grades in elementary school. Although both of these grades attend elementary school, however, the developmental characteristics of the lower grade students are partly overlapped by those of the kindergarteners. Their behavior could include a wide spectrum from light conflict to severe violence. There also needs to be an academic consensus about the term for bullying among young children in South Korea.

Intervention policies and programs should reflect a non-punitive approach (i.e., a restorative approach) and include knowledge and skills to understand their motives for bullying, and their developmental and psychological status. Further studies are necessary to develop this.

(3) Governmental support for prevention

Many findings noted in this study require government support and public awareness; interventions may involve increasing counseling centers, teachers, and supervisors, awakening people’s attention regarding the issue, and developing intervention program for teachers, parents, and children. Social support should help families and create a friendly and generous atmosphere among the parents. Prevention and intervention regarding bullying is an issue to be addressed by the whole community. Fortifying interventions does not necessarily lead to a reduction in bullying incidents. Proactive strategies may be the best solution for reducing bullying. The number of preventive services should be increased. Social emotional, or moral education are not optional, but necessary. Additionally, this should be arranged as part of the formal curriculum, meaning that preventive education will be provided on a regular basis. As Smith indicated, whatever the intervention policy, sanctions, and actions, the anti-bullying action should be effective, and for effectiveness, sustainability is truly essential [48].

## 5. Limitations

There are limitations in this study. First, the teachers’ perspectives were not included, which may be helpful for understanding why some teachers were considered incompetent by the interviewed mothers. Comparisons of the perceptions between teachers and mothers could also be useful to develop training programs for parents and teachers. Further study is needed to clarify how teachers perceive situations of young children’s bullying.

Second, the results should be carefully considered for generalization because this study was conducted with a limited sample and within a limited cultural context. This study did not investigate the fathers’ perceptions. Future research is required to understand the father’s perspectives and influences. Additionally, many participants in this study were relatively highly educated (college or higher). Although this was not intended by the authors, this may partly be caused by snowball sampling; the mothers introduced other mothers that they knew. This might have increased the similarity among the participants. Moreover, it is not clear whether the mothers’ dissatisfaction and difficulties in this study can be found in other cultures. Therefore, a cross-cultural study would be useful for investigating whether the findings are similar in other cultures.

Third, cyberbullying among young children was not included in this study. Most children aged four to eight years old have the Internet access at home and the majority of them use computers for writing letters or chatting in The Netherlands [60]. In South Korea, 51% of children under nine years old have their own mobile phone and 85% of children under nine years of age used a smartphone or computer with access to the Internet [61].

Research on the parental awareness of young children’s cyberbullying is particularly important as cyberbullying can occur over smartphones, so the parents’ role in closely monitoring their children’s use of technology is important [62]. Therefore, investigating the parental perception of use of technology or cyberbullying by young children is anticipated.

## 6. Conclusions

Acts and laws directly reflecting school bullying and prevention/intervention systems at a national level have been established in South Korea. However, the balance between the public intervention system and school ownership for dealing with bullying incidents should be considered. Systematic and public support is necessary, and long-term interventions are needed. Smith highlighted that continuing pressure on schools and policy makers is necessary to reduce bullying. Otherwise, other priorities than reducing bullying may be present, and some pressure could come from parents [48] (p. 422).

This study showed the importance of parental involvement through their voices, and emphasized the collaboration between homes, schools, and society. Additionally, it proposed a direction of intervention for young children’s bullying. Finally, it may provide information for cultures with similar circumstances to that in South Korea where bullying interventions for young children have not been researched thoroughly.
